# Characteristics of secondary epiretinal membrane due to peripheral break

**DOI:** 10.1038/s41598-020-78093-9

**Published:** 2020-11-30

**Authors:** Geun Woo Lee, Sang Eun Lee, Sun Hyup Han, Sang Jin Kim, Se Woong Kang

**Affiliations:** Department of Ophthalmology, Samsung Medical Center, Sungkyunkwan University School of Medicine, #81 Irwon-ro, Gangnam-gu, Seoul, 06351 South Korea

**Keywords:** Diseases, Signs and symptoms

## Abstract

This study aimed to investigate morphological differences between idiopathic epiretinal membrane (ERM) and secondary ERM due to peripheral break (SEPB) and to identify clinical characteristics in eyes with SEPB to facilitate peripheral retinal examination. The retrospective cross-sectional study involved 93 consecutive eyes in 91 patients who underwent ERM removal surgery. Eyes were divided into two groups: the macular pucker group and the idiopathic ERM group. En-face Optical Coherence Tomography (OCT) images, fundus photographs, severity of metamorphopsia (M-score) and clinical characteristics of each group were compared. ERM extent and eccentricity (ratio of the shortest and longest distances from the foveal center to the boundary) were obtained through en-face OCT imaging. Fundus photographs were used to judge whether the membrane was turbid or not. Patients with SEPB were younger than patients with idiopathic ERM (61.3 ± 7.5 vs. 66.6 ± 8.3 years; p < 0.05). Preoperative M-score and myopic refractive error, axial length were also significantly higher in the macular pucker group than in the idiopathic ERM group (all p < 0.05). There was no difference in ERM extent between the two groups. The incidence of ERM eccentricity was 23 of the 34 eyes (67.6%) in the SEPB group and 26 of the 59 eyes (44.1%) in the idiopathic ERM group (p < 0.05). The incidence of turbid ERM was 18 of the 34 eyes (52.9%) in the SEPB group and 10 of the 59 eyes (16.9%) in the idiopathic ERM group (p < 0.01). The SEPB group, compared with the idiopathic ERM group, tended to have eccentric, turbid ERM at a younger age and with more severe metamorphopsia and myopic refractive error.

## Introduction

Epiretinal membrane (ERM), a fibrocellular proliferation that develops on the surface of the internal limiting membrane (ILM), results in structural changes in the macula that can lead to metamorphopsia and decreased visual acuity^1^. ERM can be divided into two categories: idiopathic and secondary. Secondary ERM is associated with diabetic retinopathy, vascular disorders, retinal detachment surgery, retinal break, and inflammation, and others^2,3^.

With increased life expectancy and advances in diagnostic techniques, the prevalence of ERM is increasing^4^. Therefore, there is a growing interest in diagnosis and surgery for ERM. We can easily observe the retina without mydriasis using fundus photographs and accurately diagnose ERM using optical coherence tomography (OCT). Recently, faster and better quality OCT images have become possible, and en-face OCT images can be used to evaluate the appearance of the retinal layer. As the precise examination of the peripheral retina through mydriasis may be neglected, secondary epiretinal membrane due to peripheral break (SEPB) can be mistaken for idiopathic ERM. Neglecting a peripheral retinal break and omitting treatment of it may eventually lead to the serious complication of retinal detachment^5^.

Morphologic biomarkers of SEPB would be very helpful clinically. However, to the best of our knowledge, there have been few studies comparing the shape of SEPB with that of idiopathic ERM. The purpose of this study was to investigate the morphological differences between idiopathic ERM and SEPB, using en-face OCT images and color fundus photographs with the goal of identifying clinical characteristics of SEPB.

## Patients and methods

### Subject selection

This retrospective observational study was performed at a single center. This study approved by the Institutional Review Board of the Samsung Medical Center (IRB No.2019-04-178), which waived the written informed consent because of the study’s retrospective design and was conducted in accordance with the tenets of the Declaration of Helsinki.

A retrospective review of medical charts was done for patients who underwent pars plana vitrectomy for ERM removal between January 2018 and September 2018 by a single retinal surgeon (S.W.K) at Samsung Medical Center in Seoul, South Korea. ERM was diagnosed clinically by fundus examination by slit-lamp examination with a 90 diopter lens and spectral-domain optical coherence tomography (SD-OCT). The patients were divided into two groups: the SEBP group and the idiopathic ERM group. The idiopathic ERM group was selected by ruling out secondary ERM associated with a history of trauma, retinal detachment surgery, uveitis, diabetic retinopathy, retinal vein occlusion or other underlying maculopathy. We excluded patients with cataracts of grade II of higher (Emery–Little classification) to minimize the impact of cataracts on visual outcome^6^. All subjects underwent a comprehensive preoperative evaluation, including best corrected visual acuity (BCVA), refractive error, anterior segment examination using a slit lamp, dilated fundus examination, fundus photography, SD-OCT (Spectralis HRA-OCT; Heidelberg Engineering, Heidelberg, Germany) and en-face OCT imaging (either Triton DRI swept-source OCT; Topcon; Tokyo or Spectralis HRA-OCT; Heidelberg Engineering, Heidelberg, Germany). Decimal visual acuity was measured by using the Snellen chart and BCVA was measured by manifest refraction. All BCVAs were converted to the logMAR scale for analyses. Preoperative metamorphopsia was evaluated using the M-chart (Inami Co., Tokyo, Japan) according to a previously described method^7,8^. Metamorphopsia scores (M-scores) for horizontal and vertical lines were measured separately and the mean of the two scores was used for the analyses^9^. Routinely, all patients were hospitalized the day before surgery, and they received an extensive dilated fundus examination of the peripheral retina.

### Surgical procedure

A 23-gauge, 3-port standard pars plana vitrectomy system (Constellation; Alcon Laboratories Inc., Fort Worth, TX, USA or Associate; Dutch Ophthalmic Research Center, Inc., Zuidland, The Netherlands) was used by a single surgeon (S.W.K.). After core vitrectomy, the posterior hyaloid membrane was removed using a vitreous cutter. ERM peeling was conducted using end-grip forceps (Alcon Laboratories Inc.). ILM peeling was performed by staining with 0.03% indocyanine green (ICG) dye and using end-grip forceps for membranectomy. In most phakic patients over 55 years old, cataract extraction with phacoemulsification and posterior chamber foldable intraocular lens implantation was performed as a combined procedure of pars plana vitrectomy.

### Postoperative evaluation

Patients were followed up for at least 6 months after surgery. Follow-up examinations were performed at 1 week, 1 month, 3 months, and 6 months after surgery. Slit-lamp examination, dilated fundus examination with a 90 diopter lens, OCT, BCVA, and intraocular pressure measurement were performed at each visit.

### Outcome measures

Outcome measures were basic patient demographics and morphologic characteristics of ERM according to the presence of SEPB. Basic demographics included age, sex, axial lengths, M-score, and BCVA measured at baseline. BCVA was also serially compared at baseline, postoperative 1 month, 3 months, and 6 months. Morphologic characteristics of ERM included extent, eccentricity, and color. En-face OCT images were used to examine ERM extent and eccentricity. Fundus photography was used to evaluate turbid traits of ERM.

### Data collection

En-face OCT images were acquired in all participating patients. Automated SD-OCT software embedded in each device was used to identify and delineate separate retinal layers, including ERM, ILM, and retinal pigment epithelium (RPE) layers. Images were converted into quality-preserving JPEG images and analyzed by two independent observers (G.W.L., S.E.L.) blinded to patient information. We used open source Image J software (version 1.51; National Institutes of Health, Bethesda, MD, USA; http://imagej.nih.gob/ij/) for the imaging analysis.

**Figure 1:**
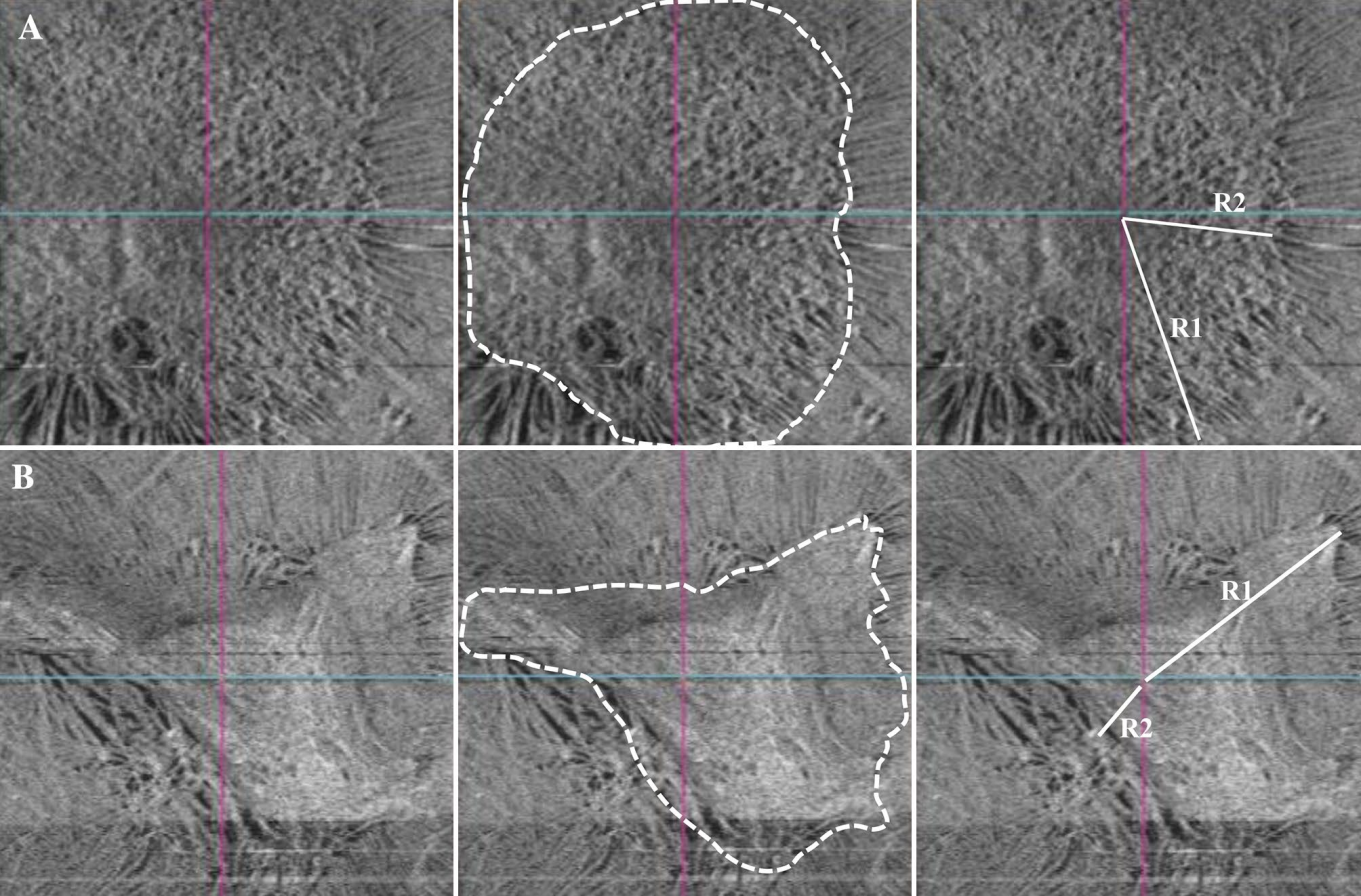
Measurement of epiretinal membrane extent and eccentricity in an en-face OCT image. Epiretinal membrane extent was defined as the area of homogenous grayish discoloration with irregular boundaries that is distinguishable from the surrounding retinal tissue on en-face OCT imaging. ERM eccentricity was measured by calculating the ratio between the longest straight distance (R1) and the shortest straight distance (R2) from the foveal center to the outer boundary of the ERM. When the ratio was equal to one (R1/R2 = 1) then the ERM was considered concentric (**A**; extent = 19mm^2^, R1/R2 = 1.6). If the ratio exceeded the median of the entire group (median = 3.2), then the ERM was considered eccentric (**B**; extent = 12.3 mm^2^, R1/R2 = 4.1).

ERM extent and eccentricity were evaluated using en-face OCT because its images most closely resemble an actual dilated funduscopic examination and the exact boundaries of ERM were frequently hard to distinguish in fundus photographs. ERM extent was defined as the area of homogenous grayish discoloration with irregular borders that was distinguishable from the surrounding retinal tissue on en-face OCT imaging (Fig. 1)^10^. When the boundaries were not clear enough to distinguish from the surrounding component, B scan images were used as a reference. In our study, the eccentricity value was determined by the ratio of the longest distance (R1) to the shortest distance (R2) from the center of the fovea to the outer boundary of the ERM (Fig. 1). In this study, eccentric ERM was defined if the ratio exceeded a specific range, which was arbitrarily set as the median (3.2) among measured values ​​of all subjects.

The color of ERM was evaluated by fundus photography. If the contour of the vessels passing under the ERM could not be clearly identified in fundus photography, we considered the ERM to be turbid.

### Statistical analyses

All continuous variables are reported as mean ± standard deviation. Independent *t* tests were used to compare the difference between two groups in BCVA, refractive error, axial length, M-score, and ERM extent. To compare ERM eccentricity and color between two groups, cross-tabulation analysis (Fisher's exact test) was used. The repeatability of the measurement of ERM extent, eccentricity, and color were assessed via the intraclass correlation coefficient (ICC) and Cohen’s kappa coefficient. The mean ICC values were 0.982 (extent, 95% confidence interval 0.976–0.988), 0.986 (eccentricity, 95% confidence interval 0.979–0.991), 0.932 (R1, 95% confidence interval 0.926–0.940) and, 0.953 (R2, 95% confidence interval 0.944–0.961) respectively, which corresponded to good agreement. Cohen’s kappa coefficient was 0.785 (color, *p* < 0.001) which corresponded to substantial agreement. Statistical analyses were performed by SPSS version 25.0 for Windows (SPSS, Inc, Chicago, Illinois, USA), and a *p* value less than 0.05 was considered significant.

### Meeting presentation

Paper was presented at the Korean Ophthalmology Society Meeting 2019, Busan.

## Results

A total of 93 eyes, which had undergone pars plana vitrectomy for ERM removal, from 91 patients were included in this study (34 eyes from 34 SEPB patients, 59 eyes from 57 idiopathic ERM patients). Of the 34 eyes included in the SEPB group, 14 eyes were previously treated with demarcation laser due to peripheral retinal break(s), and 20 eyes were treated with laser after detecting peripheral break(s) on the day before surgery. In those 14 patients, the average duration from laser treatment to ERM surgery was 14.0 ± 5.5 months.

No postoperative adverse events, such as macular hole, endophthalmitis, or retinal detachment were reported during follow-up. Patient demographics and clinical data are presented in Table 1. The mean age at the time of ERM removal was 61.3 ± 7.5 years in the SEPB group and 66.6 ± 8.3 years in the idiopathic ERM group, which was significantly different (p < 0.05). In the idiopathic ERM group, 17% were under 60 years old, 47% were in their 60s, 29% in their 70s, and 4% in their 80s. In the SEPB group, 47% were under 60, 41% in their 60s, and 12% in their 70s.

**Table 1:** Baseline demographics and BCVA of Macular Pucker and Idiopathic Epiretinal Membrane Groups.

Parameters	Total (N = 93)	SEPB (n = 34)	Idiopathic ERM (n = 59)	p value
Age, years	64.7 ± 8.4	61.3 ± 7.5	66.6 ± 8.3	0.008*
Sex (M:F)	33 (35.5%):60 (64.5%)	12 (35.3%):22 (64.7%)	21 (35.6%):38 (64.4%)	1.0
Refractive error^a^, diopter	− 1.4 ± 2.1	− 2.0 ± 2.3	− 0.9 ± 1.7	0.034*
**BCVA, logMAR**				
Baseline	0.20 ± 0.20	0.21 ± 0.21	0.18 ± 0.17	0.183
POD 1 month	0.21 ± 0.21	0.22 ± 0.24	0.20 ± 0.19	0.413
POD 3 months	0.11 ± 0.13	0.12 ± 0.10	0.10 ± 0.11	0.214
POD 6 months	0.09 ± 0.13	0.09 ± 0.12	0.09 ± 0.14	0.107
**BCVA changes, logMAR**				
Baseline–POD 3 months	0.09 ± 0.18	0.10 ± 0.18	0.09 ± 0.19	0.793
Baseline-POD 6 months	0.11 ± 0.19	0.12 ± 0.21	0.10 ± 0.19	0.639
M-score^b^	0.55 ± 0.48	0.67 ± 0.55	0.45 ± 0.46	0.023*
Axial length, mm	24.1 ± 1.3	24.6 ± 1.5	23.8 ± 1.0	0.048*
Pseudophakia (%)	16 (17.2%)	5 (14.7%)	11(18.6%)	0.778

Continuous variables are reported as mean ± standard deviation.

Statistical analysis by Independent *t* test and Fisher’s exact test.

Values with an asterisk are statistically significant.

*BCVA* best-corrected visual acuity, *SEPB* secondary epiretinal membrane due to peripheral break, *ERM* epiretinal membrane, *POD* postoperative duration.

^a^Excluding patients who underwent previous refractive surgery.

^b^Average of M-chart vertical and horizontal scores.

The mean preoperative M-score was 0.67 ± 0.55 in the SEPB group and 0.45 ± 0.46 in the idiopathic ERM group, which was significantly different between groups (p < 0.05).

One of the 28 eyes in the SEPB group and 5 of the 59 eyes in the idiopathic ERM group had previously received refractive surgery (e.g., LASIK) for myopia. A comparison of refractive error ​​was performed except in patients who underwent previous refractive surgery. Refractive error in the SEPB group was found to be more myopic (p < 0.05). In the idiopathic ERM group, visual acuity improved significantly compared with baseline (0.18 ± 0.17) at 3 months (0.10 ± 0.11) and 6 months (0.09 ± 0.14, all p < 0.01). The SEPB group also showed significant improvement in visual acuity at 3 months (0.12 ± 0.10) and 6 months (0.09 ± 0.12) compared to the baseline (0.21 ± 0.21, all p < 0.01). When comparing visual acuity improvement from baseline, the SEPB group showed higher values ​​at 3 months (0.10 ± 0.18 vs 0.09 ± 0.19, p = 0.793) and 6 months (0.12 ± 0.21 vs 0.10 ± 0.19, p = 0.639) compared with the idiopathic ERM group, but the differences were not statistically significant. The mean axial length was marginally different between the two groups (24.6 ± 1.5 mm for the SEPB group and 23.8 ± 1.0 mm for the idiopathic ERM group, p = 0.048). The sex ratio and BCVA at baseline, 1 month, 3 months, and 6 months after surgery were not significantly different between SEPB and idiopathic ERM groups (all p > 0.05).

Table 2 shows the morphologic characteristics of en-face OCT and fundus photography. The mean ERM extent was 8.4 ± 4.9 mm^2^ in the SEPB group and 8.2 ± 5.7 mm^2^ in the idiopathic ERM group (p = 0.639). The ratio of ERM eccentricity was 23 of 34 eyes (67.6%) in the SEPB group and 26 of 59 eyes (44.1%) in the idiopathic ERM group (p = 0.033). The ratio of turbid ERM was 18 of 34 eyes (52.9%) in the SEPB group and 10 of 59 eyes (16.9%) in the idiopathic ERM group (p < 0.001). In addition, we performed a post hoc analysis for the power calculation. When ERM eccentricity was set as the primary endpoint, the power of this study was 95.7% (effect size 0.47, α error 0.05, total sample size 93).

**Table 2:** Morphologic Characteristics of En-Face Optical Coherence Tomography and Fundus Photography.

Parameters	Total (N = 93)	SEPB (n = 34)	Idiopathic ERM (n = 59)	p value	Odds ratio
ERM extent, mm^2^	8.3 ± 5.3	8.4 ± 4.9	8.2 ± 5.7	0.639^a^	
ERM eccentricity (above 3.2 median)	49 (52.7%)	23 (67.6%)	26 (44.1%)	0.033^b^	4.90
Turbid color	28 (30.1%)	18 (52.9%)	10 (16.9%)	0.000^b^	13.07

*SEPB* secondary epiretinal membrane due to peripheral break, *ERM* epiretinal membrane.

^a^Calculated by Independent t test.

^b^Calculated by Fisher’s exact test.

**Figure 2:**
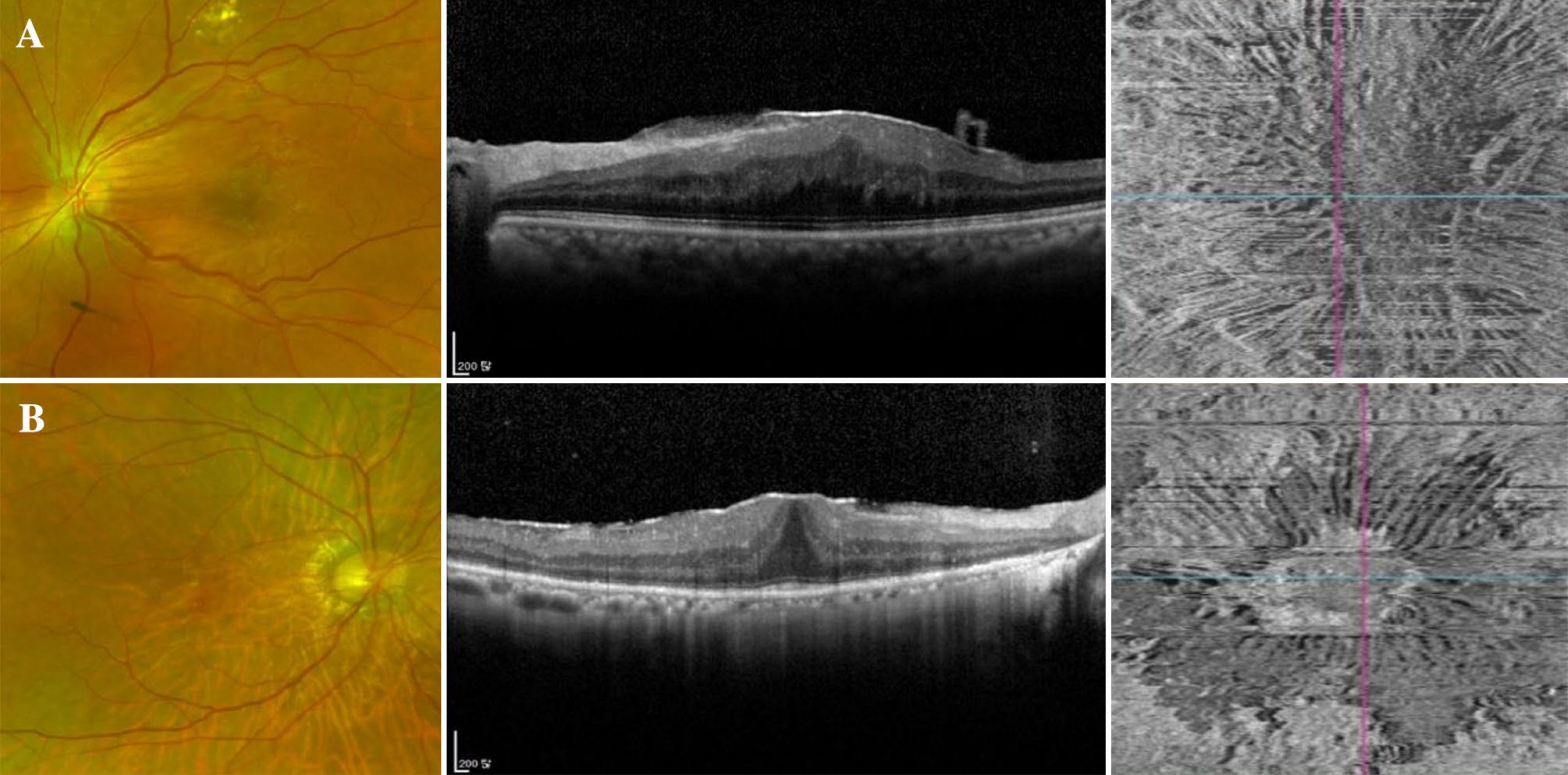
Representative cases from macular pucker (**A**) and idiopathic ERM (**B**) groups. A patient of macular pucker group (**A**) with a history of barrier laser in the past due to a retinal break showed turbidity on fundus photography and eccentricity on the en-face OCT image. R1/R2 ratio was 6.7 and ERM extent was 12.8 mm^2^. A patient of idiopathic ERM with no remarkable history showed a clear ERM on fundus photography. However, an ERM was confirmed in the OCT and a concentric appearance was seen on the en-face OCT image. R1/R2 ratio was 1.9 and ERM extent was 1.8 mm^2^.

Figure 2 illustrates representative cases of SEPB and idiopathic ERM.

## Discussion

In this study, we investigated morphological differences between idiopathic ERM and SEPB using en-face OCT images and color fundus photographs. The key findings are that (1) patients with SEPB were younger than those with idiopathic ERM, (2) eyes with SEPB showed more severe metamorphopsia and myopic refractive error, and (3) SEPB more frequently showed eccentric and turbid ERM compared with idiopathic ERM.

Most ERM is idiopathic and can be treated by ERM removal, but may be a SEPB that requires laser treatment before surgery or during surgery. If a break is found before surgery, a simple barrier laser treatment can be effective, but if it is not found early, poor prognosis can occur. Therefore, it is important to determine whether ERM is a SEPB. The peripheral retina is usually observed through preoperative mydriasis. However, if the observer does not check closely, they may miss the retinal break, which can be a risk factor for retinal detachment during or after surgery. As far as we know, no studies have examined the morphological differences between macular pucker and idiopathic ERM using en-face OCT images and fundus photography.

This study compared the clinical characteristics of SEPB and idiopathic ERM patients, and also examined morphological differences using en-face OCT images and fundus photographs. In addition to the newly proposed marker of eccentricity, color, age, preoperative M score, refractive error, and axial length ​​were significantly different between the two groups. The results of the current study indicate that SEPB, compared with idiopathic ERM, tended to have eccentric, turbid ERM, to be found in younger patients and to have more myopic refractive error and more severe metamorphopsia.

This study excluded patients with cataracts grade II or higher for accurate assessment of visual outcomes after ERM removal. The reasons are that the improvement in visual acuity after surgery may be confused with the result of cataract extraction, and to avoid the noise-induced by cataract extraction while assessing the visual outcome of ERM removal. The same reason applies to inclusion of combined cataract surgery in patients over 55 years old. In general, it is known that lens turbidity proceeds rapidly after vitrectomy^11^, and especially patients over 55 years of age. Patients over 55 years of age undergo combined surgery routinely (simultaneous vitrectomy and clear lens extraction) in this hospital.

In a previous study, their secondary ERM group was younger and had poorer visual acuity and thicker central macular thickness than their idiopathic ERM group^12^. Generally, secondary ERM is thought to occur in younger people because ERM occurs more frequently with age, and most ERM is idiopathic. SEPB may be considered to be a more severe form of ERM like the macular pucker that Gass mentioned in the past^13^. And it may be related to decreased visual acuity and severe metamorphopsia. However, the result of the current study indicates that baseline visual acuity was not different between the two groups. The relatively fair baseline vision in the SEPB group would be associated with the different patient characteristics seen in traditional secondary ERM cases after retinal detachment. That is, the SEPB group in the current study did not include the cases with macula off retinal detachment or with past history of retinal detachment surgery. Interestingly, metamorphopsia was significantly different between the two groups. The greater metamorphopsia in the SEPB group is considered to be related to the more severe ERM eccentricity in that group.

It is well known that retinal breaks and retinal detachment are more likely to occur in eyes with myopia, consistent with our findings^14–16^. Classically, idiopathic ERM formation had been thought to be related to internal limiting membrane defects induced by posterior vitreous detachment^17–19^. Recently, however, the concept of remnant cortex and hyalocytes due to anomalous posterior vitreous detachment has become more important^20–22^. ERM is developed by collagen accumulation and contractile protein production in differentiated myofibroblasts along with several kinds of cells^21,23–25^. However, in SEPB, migration, and proliferation of retinal pigment epithelial cells originating from retinal breaks are known to play an essential role in ERM development and to be more pigmented^26–30^. This fact is affirmed by our finding that the SEPB group in this study was associated with more turbid ERM. In general, SEPB is produced by random sedimentation or distribution of retinal pigment epithelial cells in eyes with posterior vitreous detachment, which may be related to the greater eccentricity of the SEPB observed in this study.

Limitations of our study include the retrospective design, limited reproducibility of manual measurements of OCT data and the fact that en-face OCT images were obtained from two different OCT machines.

In conclusion, patients with SEPB, compared with those with idiopathic ERM, tended to have eccentric, turbid ERM and were found to be younger and have more severe metamorphopsia and myopic refractive error.
